# Non-MHC Risk Alleles in Rheumatoid Arthritis and in the Syntenic Chromosome Regions of Corresponding Animal Models

**DOI:** 10.1155/2012/284751

**Published:** 2012-12-06

**Authors:** Timea Besenyei, Andras Kadar, Beata Tryniszewska, Julia Kurko, Tibor A. Rauch, Tibor T. Glant, Katalin Mikecz, Zoltan Szekanecz

**Affiliations:** ^1^Department of Rheumatology, Faculty of Medicine, Medical and Health Science Centre, University of Debrecen, Debrecen 4012, Hungary; ^2^Section of Molecular Medicine, Departments of Orthopedic Surgery, Biochemistry, and Rheumatology, Rush University Medical Center, Chicago, IL 60612, USA

## Abstract

Rheumatoid arthritis (RA) is a polygenic autoimmune disease primarily affecting the synovial joints. Numerous animal models show similarities to RA in humans; some of them not only mimic the clinical phenotypes but also demonstrate the involvement of homologous genomic regions in RA. This paper compares corresponding non-MHC genomic regions identified in rodent and human genome-wide association studies (GWAS). To date, over 30 non-MHC RA-associated loci have been identified in humans, and over 100 arthritis-associated loci have been identified in rodent models of RA. The genomic regions associated with the disease are designated by the name(s) of the gene having the most frequent and consistent RA-associated SNPs or a function suggesting their involvement in inflammatory or autoimmune processes. Animal studies on rats and mice preferentially have used single sequence length polymorphism (SSLP) markers to identify disease-associated qualitative and quantitative trait loci (QTLs) in the genome of F2 hybrids of arthritis-susceptible and arthritis-resistant rodent strains. Mouse GWAS appear to be far ahead of rat studies, and significantly more mouse QTLs correspond to human RA risk alleles.

## 1. Introduction

Rheumatoid arthritis (RA) is a polygenic systemic autoimmune disease that mainly affects the synovial joints, causing chronic inflammation and profound tissue destruction in affected patients. The pathological features of RA include leukocyte infiltration of the synovial tissue (mainly T cells and macrophages), autoantibody production (e.g., against immunoglobulins, citrullinated peptides, or tissue-restricted antigens), the accumulation of inflammatory cells (mainly neutrophils) in the joint fluid, the proliferation of synovial fibroblasts, and the formation of pannus; collectively, these features result in the destruction of articular cartilage and bone erosion. The identification of genetic alterations and variations in RA (involving either the major histocompatibility complex (MHC) or non-MHC genes) and an understanding of their functional consequences may impact the diagnosis, therapy, and prevention of RA [1], an autoimmune disease that affects approximately 1% of the human population. No other autoimmune disease appears in so many different clinical forms or is characterised by such heterogeneous and diverse clinical symptoms and laboratory tests. As a consequence, there are many experimental animal models attempting to mimic the multiple clinical symptoms of RA.

Animal studies may help to fill the gaps in human genome-wide association studies (GWAS) by allowing for gene mapping and functional studies, which cannot be performed in human patients and may yield greater insights into the mechanisms of autoimmune T and B cell responses in RA [2–4]. While the various animal models are tremendously helpful for investigating certain aspects of the human disease, none of these models recreates the full spectrum of diseases collectively called RA. Notably, thousands of investigators and pharmaceutical companies use animal models of RA, perhaps without understanding the differences among the different subtypes of this disease and the corresponding animal models [2–5]. Based upon the clinical, immunological, and genetic components, the most appropriate animal models for RA seem to be (i) those that use genetically controlled systemic autoimmune joint diseases, (ii) those in which the MHC (class II molecules) plays a crucial role, (iii) those in which both T and B cells are involved, and (iv) those that apply (auto)antigenic molecules of cartilage or joint tissues for provoking (“targeting”) synovial joint inflammation.

Among the animal models of RA that fulfil the above listed criteria from a genetic point of view and that are characterised by the presence of the most valuable biomarkers, such as rheumatoid factor (RF) and anticitrullinated peptide antibodies (anti-CCP or ACPA), the closest genetic, and clinical models of RA appear to be cartilage proteoglycan (PG) aggrecan-induced arthritis (PGIA) [6, 7] and cartilage type II collagen- (CII-) induced arthritis (CIA) [3, 8–11].

## 2. Progresses and Limitations of Human and Animal GWAS

In addition to certain MHC (or human leukocyte antigen (HLA) in humans) class-II alleles on human chromosome 6 that are most commonly (over 40%) associated with a genetic risk for RA [1, 12–16], currently there are 31 non-MHC RA risk alleles that have been confirmed by GWAS and meta-analyses [17, 18]. Many of these risk alleles are weak and are frequently “specific” for different ethnic groups or subpopulations, but there are at least 25 strong RA risk alleles within 23 non-MHC loci in the human genome that control disease susceptibility or severity [19]. These human RA risk alleles were identified and confirmed using hundreds of thousands of single nucleotide polymorphisms (SNPs) and designated by the name of the gene in which the SNP occurred most frequently. However, except for very few cases, none of the genetic risk loci identified to date represent the disease-causing or disease-promoting gene, in which mutations have occurred. SNPs, similar to postal ZIP codes, define only certain regions where a number of genes or noncoding elements (streets in the analogy) are located, but they do not define exact addresses. These risk loci or alleles defined by various numbers and frequencies of SNPs indicate only a chromosome region (carrying dozens to hundreds of genes) expected to have one or a few functionally defective genes involved in the pathomechanism of RA [20]. In fact, these RA-associated SNP risk alleles may indicate a risk for RA or a number of other autoimmune diseases [1, 19, 21–29], or their combination may be used as “predictive” markers for effective therapy selection. Due to extreme heterogeneity in the human population, the highly motivated and exciting early-stage studies have led to the current frustration, and only confirmatory or treatment-related meta-analysis studies have been published during the past couple of years. 

 In contrast to human studies using heterogeneous populations, there is a chance to use the combination of various arthritis-susceptible and arthritis-resistant inbred strains for GWAS and to identify disease-associated QTLs. There are over a hundred non-MHC genetic risk alleles identified in the rat and mouse models of RA. However, a surprisingly small number of these rodent QTLs (especially in rat arthritis models) correspond to the RA risk alleles or corresponding area in the syntenic human genomic area. Many of these rodent QTLs are listed as new discoveries and were never coordinated as the human studies were, and thus, they are frequently represented by duplicate or triplicate names when described by different research groups. Another limitation of these animal studies is that the different QTLs may represent different, probably over a dozen, phenotypes (e.g., onset, susceptibility, severity, tissue destruction, etc.) in combination with the presence or level of various biomarkers, such as autoantibodies or cytokines either in sera or *in vitro* stimulated spleen or lymph node cultures. The PCR-based method (single sequence length polymorphism, SSLP) used for the identification of QTLs in either mice or rats is a different technique from SNP microarray-based screening of the human genome, but the principal of the final linkage analysis is based on the same concept. Therefore, as it happened in human SNP-based studies where different sizes and types of arrays, populations, clinical phenotypes, disease durations, environmental factors, and responsiveness to treatment types create a heterogeneous picture of risk alleles, similar heterogeneity in genotype, phenotype, and biomarker distribution exists in animal studies.

## 3. Significance of Animal Models of RA 

Human genetic studies are expected to be fast but fairly less reliable because either the function of the SNP-identified gene or intergenic region is unknown or the consequence of the mutation found in a gene (e.g., transcription factor binding site) is very rarely known in humans. Animal studies are slow and laborious, but using appropriate genetic combinations (selected combinations of intercrosses and GWAS of F2 hybrids, congenic/subcongenic, and interval-specific congenic (IVSC) processes, and genomic sequences of the target inbred region) they can find disease-promoting genes, even with a relatively weak disease-modulating effect. Moreover, animal models allow us to investigate the role of a single gene and the mechanisms of the disease, allowing development of more effective and appropriate treatments. These animal studies, however, are valuable only if they focus on the disease-affecting/causing gene(s) in humans. Human genetics often arrives at a dead end because the disease-affecting genes are unknown [20]. Furthermore, due to the enormous heterogeneity of the human population, it is not feasible to sequence large genomic areas of thousands of people before careful selection of a relatively homogeneous subpopulation of RA patients. This selection requires extensive bioinformatics analysis comparing hundreds or thousands of disease-associated SNPs and RA patients to identify homogeneous (identical, or close to identical) SNP combinations and allele frequency for the selected RA-associated locus in affected patients. In a recent study, we compared a few hundred seropositive RA patients (all carrying the PTPN22 risk allele) but found only a dozen patients with the same SNP combinations. We expect that after high-throughput sequencing, there may be only a few (2–4) RA patients who show high genomic similarity within a small genomic region using bioinformatics analysis, but the appropriate programs and appropriate functional tests are not available at the moment.

 Although there are limitations surrounding both human and animal genome-wide screening studies, in the future, the two lines of research may support similar findings and be consolidated to provide additional insight. There are a few animal models of RA that have identified highly significant disease-associated loci. Induced autoimmune models of RA usually represent an accelerated form of RA. For example, both CIA and PGIA are known to involve MHC class-II-restricted antigen presentation and generation of T cells and autoantibodies that cross-react with self (mouse) antigens such as mouse CII or PG [3, 6, 8, 10, 30, 31]. In addition to MHC, which controls at least 40–50% of the genomic susceptibility to RA, both models require an arthritis-prone non-MHC genetic background. Nonobese diabetic (NOD) mice are resistant to both CIA and PGIA. However, when KRN T cell receptor (TCR) transgenic mice were intercrossed with NOD mice, it resulted in the K/BxN model, which develops spontaneous arthritis. The KRN TCR is specific for the bovine pancreas ribonuclease and apparently cross-reacts with glucose-6-phosphate isomerase (GPI) [32–34]. However, the spontaneous K/BxN model is irrelevant for genomic studies. It has no MHC linkage, a ubiquitous (auto)antigenic component exists (which is present in all mammalian cells [35]), and anti-GPI antibodies can rarely be detected in RA patients [36–38]. The sera of these spontaneously arthritic mice can transfer arthritis to any strain of mice (serum-transfer arthritis); thus, the genetic components of either the K/BxN or serum-transfer arthritis models are vague and unclear. However, a genome-wide screening of serum-transfer-induced arthritis in heterogeneous stock (HS) mice resulted in very interesting results [39]. QTLs identified on six chromosomes matched two human RA risk alleles (TRAF1/C5 and PADI4 loci), of which the Traf1/Hc locus on mouse chromosome 2 (mChr2) is a dominant QTL in both CIA (*mCia2 and mCia4*) and PGIA (*Pgia2*) (Table 1). 

SKG mice develop arthritis due to a spontaneous mutation in the SH2 domain of Zap70 [40]. Because the Zap70-mutation causes defective TCR signalling, it has been postulated that autoreactive T cells escape thymic deletion and accumulate in the periphery of SKG mice [40]. Altered thymic selection in SKG mice leads to the survival of otherwise negatively selected T cell clones that then spontaneously differentiate into Th17 cells in the periphery and attack the joints. In contrast, interleukin 1 (IL-1) receptor antagonist protein (IRAP) knock-out mice develop spontaneous arthritis due to increased production of proinflammatory cytokines (IL-1*β*, IL-6, IL-17, and tumour necrosis factor-alpha, TNF*α*) and autoantibodies in the absence of negative regulation of IL-1 signalling [41, 42]. In addition, human TNF*α*-expressing transgenic mice develop spontaneous chronic erosive arthritis due to their continuous production of TNF*α* [43]. This arthritis appears to be a highly simplified proinflammatory cytokine-induced arthritis; thus, it is similar to the serum transfer-induced arthritis (using anti-GPI antibody-containing sera from arthritic K/BxN mice) [44] and the collagen monoclonal antibody cocktail or LPS-induced arthritis (CAIA) [45–47].

All of these models, directly or indirectly, have contributed insights into the complex mechanisms behind RA and have facilitated the development of current therapeutics and biologics. It is important to note that all the previously mentioned experimental animal models of arthritis develop at a relatively young age (beginning at ~4–6-weeks), except PGIA [48], and that arthritis develops in SKG and IRAP-deficient mice only in the BALB/c genetic background [40–42]. This arthritis-prone BALB/c genetic background has also been shown to predispose mice to PGIA [7], human G1 domain-induced arthritis (GIA) [49], link protein [50] or human cartilage HC-gp39 protein [51]. The incidence of spontaneous arthritis in retired, breeder, wild-type BALB/c females is estimated at 0.5–1.0% (TTG, unpublished data), which is close to the ratio observed in the human population. Additionally, BALB/c mice carrying the HLA-DR4 transgene [52] or expressing a PG (5/4E8 epitope)-specific TCR [53, 54] develop arthritis spontaneously but only at an advanced age [55]. Although there are a number of other animal models of RA, we have listed only those that may have conceptual relevance to this paper. However, except for a relatively few studies [39, 56–58], GWAS in mice has almost exclusively been performed in PGIA and CIA; thus, we compare QTLs identified mostly in these two models with human GWAS and their subsequent meta-analyses (Table 1). Therefore, we summarise only those genomic regions (QTLs) of animal studies that correspond to the human chromosome region where risk alleles were identified in RA, and thus, may help to accelerate human studies. Interval-specific congenic (IVSC) mice representing human RA-associated regions present a high potential for sequencing homogeneous genomic regions, and any genes with potentially pathogenic variants (either in exons, introns or intergenic regions and in disease-promoting or disease-suppressive areas) may guide future human studies in terms of selecting appropriate patient populations for more detailed genetic and epigenetic analysis.

## 4. Tissue-Restricted (Cartilage) Antigens Can Provoke Arthritis in Genetically Susceptible Mice and May Contribute to the Severity of RA

Cartilage is one of the few immune-privileged tissues in the body in that it is essentially avascular and therefore not subjected to close “internal” immunological surveillance [59]. An incomplete central tolerance is most likely the dominant component of this special immune condition, a tolerance that can be breached when transgenes are expressed in cartilage and the cartilage-specific overexpression is “leaky,” especially in the embryo. Several lines of evidence support this hypothesis. For example, cartilage link protein [60] or otherwise arthritogenic human G1 domain (unpublished data) expression in mice, driven by the rat type II collagen promoter and enhancer, may be detected in cartilage tissue, but the transcript and protein could also be detected in other embryonic tissues. Additionally, when cartilage PG (or CII) is degraded by various matrix metalloproteinases, the newly generated neoepitopes may provoke an autoimmune reaction [61]. Further evidence is provided by posttranslational events (e.g., citrullination), as molecules unrelated to cartilage (e.g., filaggrin [62–64]) are first citrullinated far before the onset of joint inflammation. Subsequently, additional molecules (e.g., fibrinogen, vimentin, type II collagen, PG aggrecan, *α*-enolase, and a few virus proteins) also undergo posttranslational modifications (citrullination), and the cumulative effect of (auto)immune reactions may breach the immune tolerance in genetically susceptible human individuals.

 Although immunity to the cartilage PG aggrecan has been less extensively studied than immunity to type II collagen (CII), cartilage PG is also considered to be a causal factor in rheumatoid joint diseases [65–67]. Either humoral or cellular immunity, or both, to human cartilage PGs have been detected in patients with RA [65–79], and the two most recent studies reported that the citrullinated version of a dominant arthritogenic (5/4E8) peptide of human cartilage PG [80, 81] induced substantial cytokine (IL-17, IL-22, IL-6, TNF*α*, IFN*γ*) production by T cells from the majority of RA patients [78, 79]. T cells from the same RA patients responded poorly to the native (noncitrullinated) peptide in both studies, and T cells from healthy subjects did not respond [78] or responded only to the citrullinated peptide by producing IL-6 [79]. Although the majority of RA patients tested were positive for anti-citrullinated cyclic peptide (anti-CCP) antibodies (ACPA), T-cell response to the citrullinated PG peptide was also noted in some ACPA patients [78, 79].

## 5. Overlapping Genomic Loci of RA and Autoimmune Mouse Models of RA

**Figure 1:**
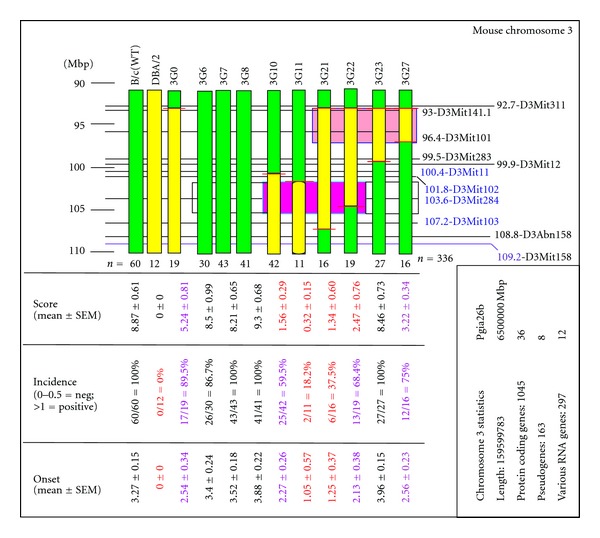
Summary of the genotypes and corresponding clinical phenotypes of parent stains and *Pgia26* (3G0) and *Pgia26* subloci that were identified in IVSC lines with overlapping chromosome intervals. The original mChr3 region (3G0: 90.4–156.5 Mbp in size) was reduced and separated into several subloci in 27 interval-specific subcongenic (IVSC) lines (3G1-3G27). For simplicity, only a 16.5 Mbp region is shown. Green columns represent BALB/c, and yellow columns represent the DBA/2 chromosome regions. Horizontal black lines with numbers at the right side (and with marker names) are shown. The short red lines crossing the IVSC chromosome region indicate the position between the two markers, where the DBA/2 allele continued as BALB/c [84]. The blue-framed red rectangular area indicates the position of the *Pgia26d* locus (between 101.4 and 107.2 Mbp); in the worst case, this region may include the entire flanking region between 99.9 and 108.8 Mbp where the disease-promoting gene(s) in BALB/c mice is located (or reciprocally, the suppressive genes in DBA/2). This area contains the most prominent *Ptpn22* (protein tyrosine phosphatase non-receptor-22) identified in human GWAS with SNPs, an allele that is associated with many autoimmune diseases. The mutation affecting R620W amino acid appears to affect both peripheral and central B-cell tolerance [120]. Under the worst scenario, this region contains 128 protein-coding genes, 19 miRNAs, 13 pseudogenes, and 9 non-protein-coding transcripts (http://www.ensembl.org/Mus_musculus/Info/Index). Other *Pgia26* subloci (with large scales) are presented in Figure 2 with the corresponding human, rat, and mouse RA risk alleles. Another disease-suppressive region (inherited from the DBA/2 strain), between 92.7 Mbp and 96.4/99.9 Mbp position (framed), is currently under sequencing and examination.

**Figure 2:**
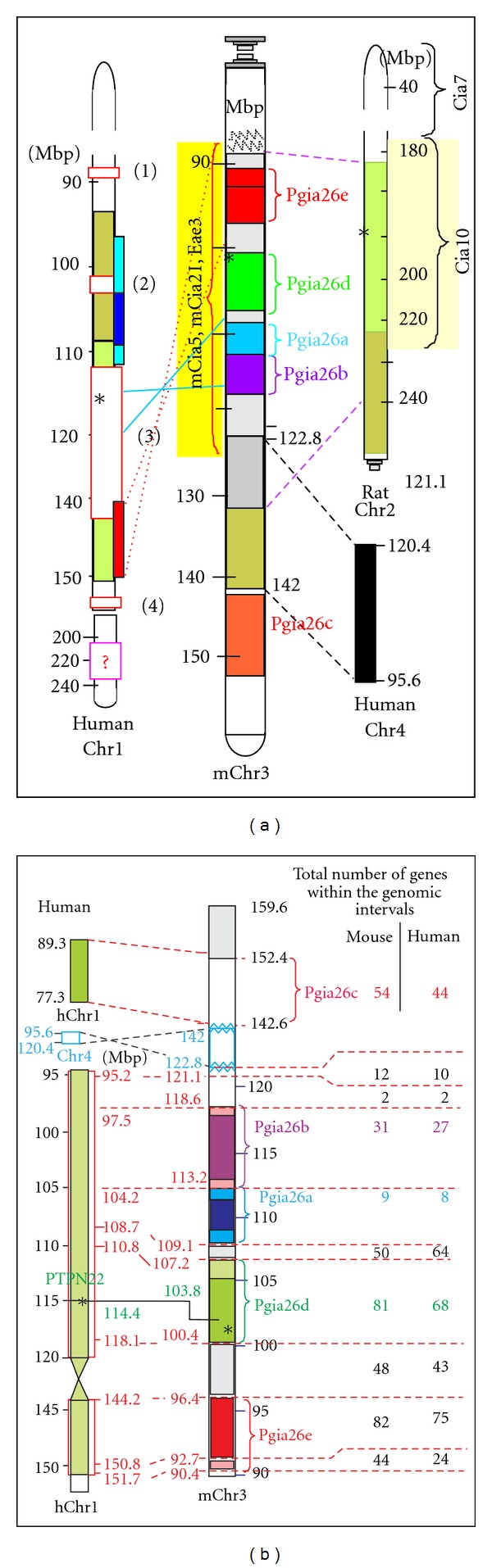
Mouse chromosome 3 (Chr3) with *Pgia26* subloci identified in IVSC mice (Figure 1) and corresponding human and rat chromosome regions with their corresponding risk alleles. Panel (a) summarises the location of five *Pgia26* subloci with corresponding mouse *mCia5 *and* mCia21 *(collagen-induced arthritis) [3, 108, 121], mouse Eae3 (experimental allergic encephalomyelitis) [122, 123] (between mChr1:84.3–126 Mbp), and the corresponding rat Chr2 region of rat *Cia10* [124, 125]. The left side of the panel identifies risk alleles on human Chr1 [126], with red-framed boxes and numbers in parentheses indicate the following regions: (1) between 87–89 Mbp [127], (2) 105.4 Mbp [128], (3) 113–142 (including the PTPN22 gene at 114.4) Mbp positions [129–131], and (4) the FCGR family between 158 and 159 Mbp [132, 133]. Panel (b) displays the syntenic risk alleles of human Chr1 and mouse Chr3 (*Pgia26a-e*) with the number of genes localized in the different chromosome regions.

In this paper, we collected results from GWAS in mice and rats (over 100 QTLs) and compared the QTL localisations to those identified in human studies (over 30 RA-associated loci). It is technically impossible and scientifically unnecessary to cite all these studies; rather, we tried to select those that represent syntenic regions in humans and mice (and rats if available). We cite the most appropriate publications in Table 1 or in the text rather than indicating SNP codes (rsXXXX). The levels of significant association between the same SNP and RA is variable in different papers, and for the novelty of a new meta-analysis, investigators may preferentially use a SNP in close proximity to those that have already been published. In brief, we selected data from RA risk allele groups that also have syntenic regions in rodent studies and show one of a few on-going animal/human studies (mouse Chr3 versus human Chr1) in which the combined information may be not only quantitative but also qualitative (Figures 1 and 2). In other words, two chromosome regions (Figure 1) have not only SSLPs (andSNPs) in the “candidate” target regions but also functional defects in the protein encoded by the mutated gene that may either suppress or promote the onset and severity of arthritis. Thus, these particular mouse studies aid in the discovery of functional defects in disease-associated genes in humans with RA.

 As mentioned, over 100 rodent QTLs have been described to date, but relatively few are syntenic with any of the 30 human RA risk alleles. In our laboratory, over 5,000 inbred wild-type parents, approximately 500 F1 hybrids (all negative for PGIA, data not shown) and 3,200 F2 hybrids of six different genetic intercrosses were genotyped using a total of 240 SSLP markers. The goal was to identify genetic alterations responsible for individual and overlapping qualitative (binary) QTLs that are linked to PGIA or CIA in the mouse genome and then compare the results with loci identified in human autoimmune diseases, preferably RA. Many of the risk alleles in RA overlap with a number of risk alleles of other autoimmune diseases [19, 21–27, 29], and a number of *Pgia *and *Cia *loci [10, 82–87] overlap with chromosomal regions identified in GWAS studies of RA patients [17, 19, 88–91]. CIA was considered as a model of seronegative RA, whereas PGIA, which has both rheumatoid factors and ACPA [7, 49], was considered a seropositive RA model. The overall hypothesis was that genes associated with a QTL in one or more genetic combinations of murine autoimmune arthritis should correspond to genes involved in RA. (A total of 26 loci out of 31 confirmed non-MHC loci were screened for corresponding mouse QTLs. Only those that were found in comparative studies of mouse genome-wide association (GWAS) studies (*n* = 17) are listed under the “human locus name.” These mouse GWAS studies include over a dozen intercrosses screened in different laboratories. Occasionally, the same (mouse) *Cia* locus-number appears on different chromosomes in different publications, thus the references corresponding to the appropriate mouse *Cia* (*mCia*) loci are listed here. QTL of *Pgia* (*n* = 9) and *mCia* (*n* = 2) identified in our laboratory are italic and bold faced. Each human locus is listed by the gene-name and chromosome location using the “standard” name of the given RA risk allele; the corresponding mouse region/gene is indicated by the same gene name and location in the mouse genome given by the mega-base pair (Mbp) position (bold-faced). Tissue samples (tails and kidney) of each F2 hybrid mouse are catalogued and stored at −80°C. Many of the F2 hybrids were retested with additional, new markers in confirmatory studies (9 *Pgia* and 2 *mCia* loci). The average marker density in these confirmatory studies was 8.2 Mbp. Some of these reference markers shifted slightly after confirmatory studies using high density marker screening. Two QTLs on mouse chromosomes 3 and 15 have overlapping regions; therefore, they are listed in the Table 1 twice due to the information from different studies.)

 Although there are a number of weaknesses for both human and animal GWAS, they may supplement and support each other. During the past 15 years, we and others have identified 29 *Pgia* and 40 *Cia* loci in different genetic combinations of F2 hybrid mice [3, 10, 11, 57, 58, 82, 86, 95–99] and a couple of corresponding QTLs in rats [100–105]. With a strong confirmation in the literature, we selected QTLs from all (published) mouse genomic studies [10, 56–58, 82–84, 92–96, 98, 106–112] that also correspond to one of the major risk loci of RA confirmed in a number of meta-analyses [19, 29, 90, 113–117]. Table 1 summarises the risk alleles selected that have corresponding genomic regions from human and mouse GWAS. Only QTLs that correspond to at least one major RA-associated locus in the human genome are listed; these QTLs were found on mouse chromosomes 1 (2x), 2, 3 (2x), 5, 6, 10 (2x), 13, 15 (2x), and 18 (a total of 13 QTLs). The list was organised in order of mouse chromosomes. At least one, and possibly two or three, QTLs from various animal studies covered the syntenic chromosome region of human RA-associated loci. Standard abbreviations of genes were used as they are listed in gene bank databases (e.g., http://www.informatics.jax.org/; http://www.ensembl.org/Mus_musculus/Info/Index or http://genome.ucsc.edu/cgi-bin/hgGateway), and many of their known functions are described in publications available from PubMed (http://www.ncbi.nlm.nih.gov/pubmed). Thus, we did not list the full names or discuss the function(s) of these genes used to identify RA susceptibility loci or the “most frequent” associated SNPs of meta-analyses. These “marker-specific” genes were usually located near the unknown genes that might carry the disease-causing genomic defect. For example, SNPs of two of the strongest RA risk alleles, TRAF1/C5 and TNFAIP3/OLIG3, are in the intergenic regions, making it difficult to establish causality of these regions at this moment [20, 88, 118]. Although both TRAF1 and TNFAIP3 are “preferential” gene candidates based on their function in TNF signalling, known to be important in RA [119], none of the genes having SNPs or genomic mutations evidently affect their function.

 In the next section, we show an example of how we can integrate information from the human and mouse studies. This method may be one of the potential ways to identify causal variants that map to human RA-associated chromosome regions.

## 6. Benefits of MHC-Matched Susceptible and Resistant Mouse Strains: IVSC Strains Targeting Human RA Risk Alleles

To eliminate or reduce the dominant effect of MHC in cases where the association of a QTL with an arthritis phenotype has been sufficiently confirmed, one of the most successful alternative approaches is to use MHC-matched arthritis-susceptible and arthritis-resistant strains to establish congenic and subcongenic lines. Either a disease-promoting chromosome region can be “inserted” into a resistant strain, or reciprocally, the same region containing a disease-suppressive allele can be inserted into a fully susceptible genetic background. Either direction is acceptable, but from a practical point of view and based on many congenic experiments during the past decade, the latter solution appears to be more manageable. First, F1 males are selected, for example, from the intercross of a PGIA-susceptible BALB/c female and a resistant DBA/2 male (both MHC H2^d^) carrying the DBA/2 genomic region of interest. These F1 males are backcrossed several times with wild-type BALB/c females, and the offspring are genotyped for each litter until the N_1_-N_X_ generations have sufficient numbers of recombination events (and, if possible, overlapping areas) (Figure 1). These N_x_ males are intercrossed with wild-type BALB/c females, and the resulting heterozygous N_x−1_ males and N_x−1_ females are intercrossed to establish a homozygous IVSC strain(s).

During the ongoing backcrossing process, fewer and fewer previously heterozygous loci need to be tested by PCR. If a gender effect is expected, it is necessary to replace the Y chromosome with a single reciprocal backcross, but it is both practical and sufficient to do this replacement near the final step.

Subsequently, the chromosome intervals from the resistant strain of a relatively (and usually) large QTL (several cM or Mbp in size) need to be tested for clinical phenotypes. For example (Figure 1), the “Chr3G0” (“3G0”) subcongenic line contains an overlapping region ~66 Mbp in size that significantly affected all clinical phenotypes when compared to either susceptible BALB/c or resistant DBA/2 parental strains [84], a finding that needs to be further confirmed by *in vitro* tests (i.e., measuring biomarkers). In this case, males from the congenic 3G0 strain can be used to reduce the chromosome interval with new recombination events with matings into inbred BALB/c females. On the other hand, only the critical interval of mChr3 with high-density markers needs to be genotyped because the entire genome was previously genotyped for BALB/c (during the selection of 3G0 congenic line). Then, mice with the most appropriate recombination products are used as founders for fine mapping of chromosome intervals generating IVSC strains. Conceptually, the same backcrossing to the susceptible BALB/c strain and genotyping approach, as described above, are used for the selection of new congenic strains. However, investigators need to (i) focus on the new recombination events within selected chromosome interval using high marker density within the region of interest (e.g., *Pgia26*) and (ii) genotype both males and females. Depending on the volume of backcrossing (i.e., the number of breeding pairs and offspring) and the shortest chromosome interval achievable after a few generations, we are able to select a number of heterozygous males and females with identical recombination events at different positions (if possible with overlapping regions as shown in Figure 1: e.g., Chr3G0-Chr3G27) to establish homozygous IVSC strains for *in vivo* and *in vitro* tests.

 To save time, it is practical to genotype both males and females for all new recombination products within the chromosome interval of interest, a locus that corresponds to the selected human RA risk allele. As shown in Figures 2(a) and 2(b) and Table 1, the *PTPN22/CD2 *human risk locus most likely represents a complex trait on mChr3 (syntenic with hChr1) containing both disease-suppressive and disease-promoting alleles [84]. Distinct regions, alone or in combination, may result in clinically similar phenotypes (Figure 1), while the IVSC-associated biomarkers may show significant differences. Thus, a relatively small IVSC chromosome region may be separated for different genotypes representing similar clinical phenotypes (Figure 1, only the centromeric region of the mapped mChr3 is shown). However, while clinical phenotypes are comparable, fundamentally different genes in nearby chromosome regions may control disease susceptibility, onset and severity. Needless to say, fine mapping of chromosome regions and selecting narrow genomic regions with high probability for successful genomic high-throughput sequencing might be difficult, if not impossible, to complete using RA patients from the heterogeneous human population. Further, this highly specific and laborious animal study is valuable only if it represents human relevance, that is, if the corresponding region where the human risk allele was localised had already been identified.

Figure 2 shows simplified schematics comparing the previously outlined IVSC approach (*Pgia26* on mChr3) in combination with mouse (*mCia*) and rat CIA loci syntenic with the RA loci identified on human Chr1. Colours, numbers of genes, locations of syntenic genomic loci, and their flanking regions are indicated in Figure 1 and legend. With the advent of genome sequencing techniques, SureSelect Target Enrichment kit (Agilent, San Diego, CA, USA), library amplification and Illumina parallel sequencing methods made it realistic to oversequence 10–30 Mbp of homogeneous genomic regions from inbred IVSC strains and compare sequences with parent strains (susceptible versus resistant). It is also a reasonable approach to confirm the function of arthritis-susceptible or arthritis-resistant murine strains with transgenic methodologies. Today, the real challenge in human genetics is to find and select appropriate human patients with nearly identical genomic region(s) for high-throughput genomic sequencing due to the extreme heterogeneity of the human population. While SNP analyses using thousands of samples can give an extremely high statistical power, the same approach (SNP selection for genomic sequencing) is unsuccessful in the selection of human samples [20].

However, there are promising directions based on the combination of human-mouse GWAS. Selected homozygous regions of IVSC mice sequenced first with high-throughput sequencing method and affected genes and/or intergenic (relatively small) regions are genome-sequenced from selected humans with appropriate primers. In fact, a certain number of mutations/SNPs of the syntenic regions (identified in IVSC mouse and confirmed using conventional Sanger sequencing of human genomic DNA) may guide the selection of human RA patients for high-throughput sequencing of the region of interest (Figure 2). Alternatively, for example, if miRNA-related sequences are expected, the high-throughput sequencing of RNAs isolated by cross-linking immunoprecipitation (HITS-CLIP) with antibodies against the RNA-binding protein Argonaut (Ago HITS-CLIP) [134–138] may offer another solution.

## 7. Overall Summary and Perspectives

Overall, mouse studies, especially with congenic strains, appear to be a fundamental resource for the identification of candidate gene(s) in RA. During the past 15 years or so, almost concurrent with the first human genomic studies in RA, a number of rodent (mouse and rat) GWAS studies have been performed. At approximately the same time, both the human and mouse genome sequencing studies were completed and, simultaneously, unlimited numbers of new markers became available for both species. The number of human studies expanded; tens of thousands of RA patients, along with controls, were genotyped; new and more reliable SNP arrays became available; more risk alleles became identified in RA and in almost all autoimmune diseases. However, after extensive progress in GWAS, the direction of RA research moved towards confirmatory studies of previously tested patients, examinations of different ethnic groups or comparisons of the therapeutic effects of different biologics. Briefly, human studies turned to mainly *in silico* and meta-analysis studies rather than aimed towards finding causative and functional (aetiological) reasons. The previously identified genomic regions were confirmed using a high marker density, but the large chromosome regions with tens of Mbps in size still remained unmanageable. Only a very few SNPs causing missense mutations proved to be associated with disease, and usually only in a narrow selection of the patient population. However, the number of risk alleles increased, and previously identified marker positions were confirmed.

Unfortunately, animal studies also slowed down, although due to completely different reasons from human studies. Increasing the number of new combinations of disease-susceptible and disease-resistant inbred strains revealed more and more QTLs, but not a disease-causing gene. Recognising the limitations as well as the potential of both human and mouse GWAS, approximately 10 years ago, a number of congenic strains carrying the most promising traits representing the strongest clinical phenotypes were established. These strains carry overlapping traits identified in different animal models and syntenic with genomic regions identified as RA risk alleles. In other words, at the time when the human GWAS explored the most critical RA risk alleles, congenic backcrossing had selected inbred IVSC strains with syntenic regions to the major human risk alleles. We selected two QTLs for more detailed analysis: *Pgia26/Cia5/mCia21/Eae3* on mChr3 and rat *Cia10,* corresponding to the *PTPN22/CD2* allele on human Chr1 (Figure 2); *Pgia2/Cia2/Cia3 *on mChr2 (corresponding to the* TRAF1/C5* allele on hChr9). Then, we generated IVSC strains (Figures 1 and 2, *Pgia26* is shown). All other congenic and subcongenic strains were cryopreserved. The two major/dominant mouse QTLs were separated into narrow subtraits and simultaneously tested for arthritis susceptibility, for disease onset and severity, and for over 15 biomarkers that might have some potential relevance for RA [84]. Simultaneously, some of the IVSC genomic regions representing homogeneous regions of disease-susceptible and disease-resistant IVSC mice (and the corresponding parent genomic regions) were sequenced, and a few mutated genes were identified (with “known” or completely unknown function). Occasionally, these genes had not been previously associated with arthritis, but all of them had localised in close proximity to a gene used to name the human RA risk alleles. The analyses of these genes and a targeted selection of appropriate human genomic DNA samples used for high-throughput sequencing are currently in progress in a number of laboratories. The approaches and concepts outlined in this paper (especially in Sections 4 and 5) are not the only possible avenues for the identification of the RA (or other autoimmune disease)-related defects in the genome. However, these approaches may allow us to merge currently available results of human GWAS with findings of GWAS and IVSC studies in mice. Nonetheless, to confirm the role of these genes in RA, researchers must identify not only the genomic identity but also the corresponding functional defects in mice analogous with those present in patients with RA. Unfortunately, mechanistic and functional studies, manipulation of the genome, and pretesting of new therapeutic approaches cannot be applied in human patients, which underlines the relevance of and necessity for laborious genetic studies in animal models.

## Figures and Tables

**Table 1:** Arthritis-associated QTLs of mice corresponding to human SNP-based GWAS.

mChr	Markers	Region (Mbp)	Locus name (mouse)	Peak marker name	Peak marker position	Mouse gene And its position (Mbp)		References (mouse)	Corresponding human Chr position (Mbp)		Human locus name
1	D1Mit188– D1Mit122	3.9–40.5	***mCia14 ***	D1Mit244	37.6	**Aff3**	**(38.2)**	[9, 40, 92, 93]	**2q11**	**(100.2)**	**AFF3**
1	D1Mit426– D1Mit209	90.4–193.3	***mCia9 *** ***Pgia1 ***	D1Mit166	178.3	**Fcgr2b**	**(170.9)**	[57, 92, 93]	**1q23**	**(172.9)**	**FCGR2A**
			*Pia(Chr1) *	D1Mit36	171.1						
2	D2Mit79– D2Mit237	21.9–40.8	*mCia2* *mCia4 *	D2Mit238	33.9	**Traf1** **/Hc**	**(34.8)**	[2, 40–44]	**9q33**	**(123.6)**	**TRAF1/** **C5**
			***Pgia2 *** HS	D2Mit81	33.4						
3	D3Mit75– D3Mit284	100.5–103.6	*mCia21* *mCia22 *	D2Mit75 D2Mit284	100.5 103.6	**Cd2** **Ptpn22**	** (100.5)** **(103.6)**	[45, 46]	**1p23** **1p13**	** (114.3)** **(117.3)**	**CD22** **PTPN22**
3	D3Mit141.1–D3Mit323	93.0–152.4	***Pgia26 *** ***(+3 subloci) ***	D3Mit158	109.1	**Cd2** **Ptpn22**	** (100.5)** **(103.6)**	[47, 47, 48, 57, 92, 93]	**1p13** **1p23**	** (114.3)** **(117.3)**	**CD22** **PTPN22**
			*Pia*-Chr3	D3Mit100	96.8						
5	D5Mit388– D5Mit256	33.8–63.3	***Pgia16*** *mCia13 *	D5Mit233	53.0	**Rbjp**	**(53.9)**	[9, 44, 49]	**4p15**	**(25.8)**	**RBJP**
6	D6Mit86– D6Mit318	4.4–65.1	***Pgia19 ***	D6Mit267	29.2	**Irf5**	**(29.4)**	[40, 50, 93]	**7q32**	**(128.2)**	**IRF5**
10	D10Mit206– D10Mit130	13.8–65.9	***Pgia6 *** ***Pgia6b ***	D10Mit124	20.9	**Tnfaip3** **Prdm1**	** (18.7)** **(44.1)**	[9, 40, 43, 92, 93]	**6q23** **6q21**	** (138.2)** **(106.5)**	**TNFAIP3** **PRDM1**
10	D10Mit12– D10Mit269	98.9–128.4	*mCia8* (“locus 5”) *AIL(Chr10*)	D10Mit102 D10Mit261	120.3 85.0	**Kif5a** **Pip4k2c**	** (126.7)** **(126.6**)	[40, 50, 58, 58, 94]	**12q13** **12q13**	** (57.9)** **(57.9)**	**KIF5A** **PIP4K2C**
13	D13Mit258– D13Mit78	95.6–119.6	***Pgia15*** *mCia19 *	D13Mit51 D13Mit53	105.3 113.1	**Ankrd55** **IL6st**	** (113.0)** **(113.2**)	[9, 44, 51, 92, 93]	**5q11** **5q11**	** (55.4)** **(55.2)**	**ANKRD55** **IL6ST**
15	D15Mit121– D15Mit242	57.9–90.2	***Pgia9 ***	D15Mit28	74.4	**IL2b** **Bik**	** (78.3)** **(83.4)**	[9, 40, 44, 62, 92, 93]	**22q12** **8p3**	** (37.5)** **(11.3)**	**IL2RB** **BIK**
15	D15Mit279– D15Mit192	74.0–92.7	*mCia35–mCia37 *	D15Mit159	87.3	**IL2b** **Bik**	** (78.3)** **(83.4)**	[53]	**22q12** **8p3**	** (37.5)** **(11.3)**	**IL2RB** **BIK**
18	D18Mit51– D18Mit80	61.3–77.0	***Pgia11 ***	D18Mit81	66.7	**Ptpn2**	** (67.8)**	[9, 40, 43, 93]	**18p11.3–p11.2**	**(12.8)**	**PTPN2**

Human RA loci validated in Caucasian, African American, and Asian ancestry and compared via meta-analysis [17, 18, 54, 55, 59–61, 63].

Human RA loci validated in Caucasian, African American, and Asian ancestry and compared via meta-analysis [17, 18, 54, 55, 59–61, 63].

## Abbreviations:

Anti-CCP: Anti citrullinated peptide antibody
ACPA: Anti-citrullinated protein antibody
CII: Type II collagen
CAIA: Collagen antibody-induced arthritis
CIA: Type II collagen-induced arthritis
Chr: Chromosome
cM: Centi-Morgan
GIA: Human cartilage PG's G1 domain-induced arthritis
GPI: Glucose-6-phosphate isomerase
GWAS: Genome-wide association studies
HLA: Human leukocyte antigen
HS: Heterogeneous stock
IFN*γ*: Interferon gamma
IL: Interleukin
IVSC: Interval-specific congenic
*mCia*: QTL of mouse CIA
Mbp: Mega-base pair
MHC: Major-histocompatibility complex
QTLs: Quantitative trait loci
PG: Cartilage proteoglycan aggrecan
PGIA: PG aggrecan-induced arthritis
*Pg**ia*: QTL of PGIA
*P**ia*: QTL of pristane-induced arthritis
RA: Rheumatoid arthritis
RF: Rheumatoid factor
SSLP: Single sequence length polymorphism
TCR: T cell receptor
TNF*α*: Tumor necrosis factor alpha.
